# Factors facilitating trained NIMART nurses’ adherence to treatment guidelines: a vital matter in the management of TB/HIV treatment in South Africa

**DOI:** 10.1186/s12912-020-00470-6

**Published:** 2020-08-17

**Authors:** Lufuno Makhado, Mashudu Davhana-Maselesele, Rachel Tsakani Lebese, Sonto Maria Maputle

**Affiliations:** 1 Department of Public Health, School of Health Sciences, University of Venda, Thohoyandou, South Africa; 2 Department of Nursing Science, Faculty of Health Sciences, University of Pretoria, Tshwane, South Africa; 3 Research office, School of Health Sciences, University of Venda, Thohoyandou, South Africa; 4 Department of Advanced Nursing Science, School of Health Sciences, University of Venda, Thohoyandou, South Africa

**Keywords:** Adherence, NIMART, NIMART-trained nurses, TB/HIV, Treatment guidelines

## Abstract

**Background:**

Globally, the burden of tuberculosis or human immunodeficiency virus (TB/HIV) is at 24% and this alarming rate compelled the World Health Organization (WHO) to declare the African region as a critical workforce shortage area. To facilitate adherence to treatment guidelines, WHO recommended a strategy of task shifting for countries with high health workforce shortages. The strategy aimed at the redistribution of health care tasks to available workers. The study aimed to determine the factors facilitating nurse-initiated management of antiretroviral therapy (NIMART) trained nurses’ adherence to TB/HIV treatment guidelines.

**Methods:**

The study employed an exploratory-descriptive design. The study was conducted in Ugu and Ngaka Modiri Molema Districts of KwaZulu-Natal (KZN) and North West (NW) Provinces of South Africa. The population comprised of 24 participants who were purposively selected. The in-depth focus group discussions were conducted and ATLAS T.I. was used for data analysis following the basic steps of notice-collect-think (NCT) analysis. Trustworthiness and adherence to ethics were ensured.

**Results:**

The singular theme of factors facilitating NIMART trained nurses’ adherence to treatment guidelines which included positive attitudinal needs and positive behavioural change emerged from raw data.

**Conclusion:**

Continuous training, support supervision, and improved relationships with colleagues need to be enhanced to enable NIMART trained nurses to adhere to treatment guidelines.

## Background

Globally, the burden of tuberculosis (TB)/ human immunodeficiency virus (HIV) disease is at 24%, with a 3% global health workforce that compelled the World Health Organization (WHO) to declare the African region as a critical workforce shortage area [1]. To facilitate adherence to treatment guidelines, WHO recommended a strategy of task-shifting for countries with high health workforce shortages [2–4]. The task-shifting strategy aimed at the redistribution of health care tasks to health care practitioners that are available. With severe shortages of physicians and the increasing burden of TB/HIV co-infection and pandemic, there was an increased demand for access and adherence to antiretroviral treatment (ART) [5]. This initiated the shifting of ART initiation and management from physicians to nurses. There has been a lot of evidence about the growing shifting of these ART tasks to nurses in African countries, but little is known about the impact of implementing this model and adherence to treatment guidelines on nurses on the African continent [6].

Task shifting is an old phenomenon; in France and China substitutes for physicians has been a practice as far back as the nineteenth century [7]. In African countries, training of non-physician nurses was done for different roles with good health outcomes although there are critics who suggest that task shifting performed uncritically at the expense of health workers often leads to low salary, poor working conditions and high attrition [8]. A concern was therefore raised that shifting additional HIV tasks to lower health workers categories could risk competing with other service priorities, especially the adherence to treatment guidelines [8]. The World Health Organization (WHO) published recommendations to promote the initiation of ART for all persons with TB/HIV co-infection, irrespective of the CD4 cell count [9].

Management of TB/HIV relies on the proper implementation of guidelines by NIMART trained nurses. Precise and systematic implementation of treatment guidelines is critically important if the TB/HIV treatment outcomes are to be realised. Implementation of ART initiation have been undertaken at specific points of time in South Africa. Before 2009, South Africa’s treatment guidelines reserved initiation of antiretroviral therapy (ART) for adults with CD4 counts < 200/mm3 [10]. In 2010, the revised hints outlined eligibility for ART amongst individuals with TB/HIV to consist of those with CD4 cell count 350 cells/mm3 and those with multidrug-resistant or extensively drug-resistant TB (MDR-TB or XDR-TB), irrespective of CD4 cell count [11]. The suitable TB case management, and provision of all-inclusive HIV care to TB/HIV co-infected patients, is consequently vital in prolonging the lives of people living with HIV (PLWH), as well as reducing the negative effects of TB on the course of HIV treatment whilst interjecting the potential spread of TB.

The National Department of Health developed a National Strategic Plan for HIV, STIs and TB 2017–2022 to respond to the dual epidemics [12, 13]. The focus of this study is on HIV and TB, and professional nurses (PN) training on NIMART programme to provide comprehensive TB/HIV management. Since adherence to treatment is a key determinant of TB/HIV treatment outcomes, the use of facilitators in the implementation of guidelines should be promoted [14]. The evidence highlights that nurses generally display a more positive attitude than physicians in the implementation of the clinical practice guidelines (*p* < 0.001) [15, 16]. Also, their negative mentalities about the significance and absence of inspiration in utilizing clinical practice guidelines were identified with diminished use [15, 16]. Furthermore, nurses resistant to change and lacking in motivation and commitment towards adhering to the use of clinical practice guidelines were less likely to use them. Professional nurses who were persuaded that clinical practice guidelines have improved and strengthened the health care system and patient results were bound to report the utilization of and adherence to them [17]. The latter was the opposite when compared to those who did not perceive the usefulness of clinical practice guidelines [17]. The literature further indicates that when nurses perceived clinical practice guidelines as useful and relevant, they were more likely to encourage other professional nurses to practice the usage and adherence to the treatment guidelines [18].

Knowledge is a facilitator for clinical practice guidelines utilization and adherence. Noticeably, introduction of information sessions through training and education during the initial clinical practice guidelines implementation as well as continuous exposure to inservice training throughout are recommended for all professional nurses determined to intensify their adherence and use thereof [18–23].

According to a study that explored barriers to treatment guidelines adherence between NIMART trained nurses in KwaZulu-Natal (KZN) and North West (NW) Provinces concluded that there is a need for South Africa to recognize, accept and address the available barriers to treatment guidelines adherence [24]. Moreover, the same study identified that TB/HIV treatment guidelines adherence is a complex encounter that calls for innovative solutions [24]. The following challenges were highlighted: the need for increased communication; the need for improved supportive supervision between organisational management and NIMART nurses; amplification of human resources to condense workload; reported attitudes of NIMART trained nurses and appropriate documentation of clinical records [24]. Hence, this paper was designed and developed to determine factors facilitating TB/HIV treatment guidelines adherence amongst trained NIMART nurses’ in the KZN and NW Provinces of South Africa.

## Methods

A qualitative approach was employed for this study using an exploratory-descriptive design to explore and describe the phenomenon of interest through eliciting the viewpoints of people most affected [24–26]. Systematic random sampling was used to select the sixteen facilities which comprised of eight Community Health Centres (CHCs) and eight Primary Health Care (PHC) facilities from KZN and NW provinces respectively. The population comprised of all NIMART trained nurses in KZN and NW Provinces of South Africa who initiate and manage ART and anti-TB treatment [24, 26]. The researchers employed convenience sampling to sample NIMART trained nurses who had 12 months and more working in CHCs or PHCs, accredited to provide ART services [24, 26]. All CHC and PHC NIMART trained nurses were recruited to participate and only 24 of them agreed to participate in the study. The researcher visited the unit as per the appointment to get the signed consent of the participants [26].

### Study setting

The study was conducted in the selected CHC and PHC facilities of Ugu and Ngaka Modiri Molema Districts in KZN and NW provinces of South Africa respectively [24, 26].

### Data collection

Focus group discussion (FGD) was used to collect data and was guided by a central question posed to the participants: ‘*What are the factors facilitating treatment guidelines adherence among NIMART trained nurses?’* which is one of the FGD guide questions from the parent study [26]. The central question was discussed while probing and follow-up questions enhanced the quality of data collected [24, 26]. All participants had the liberty of choosing the time of the day for the FGDs and as well as follow-up sessions. The FGDs were conducted in a selected hospital in Port Shepstone and North-West University School of Nursing Sciences boardroom and each lasted between 90 and 140 min [24, 26]. A digital recorder was used to document all FGDs.

Observational and field notes aided in the triangulation of data collected. Data were collected until saturation, where no new information was emerging at the fourth session of each of FGDs from both districts. Audiotaped data were transcribed verbatim for posting the discussion for further analysis.

Before commencing with data analysis, the study transcripts were matched with audiotaped data, observational and field notes to ensure the accuracy of the transcripts [24, 26]. Descriptive analysis was carried out using a demographic datasheet to describe the demographic characteristics of the participants. ATLAS.ti (7.0) was used following the Friese’s basic steps of notice-collect-think (NCT) analysis [24, 26, 27]. The researchers began by noting prominent and recurrent features of the data and this was collated following the predetermined codes [24, 26, 27]. The process included the following steps: getting acquainted with the collected raw data; generating a directory of all the codes and categories; application of the code directory on raw data through marking coding the transcripts; separating the interrelated data bearing the identical code into a different file; and inferring emerging themes from the analysed data concerning the variety and strength of the views emerging while establishing perceived relations between the themes [24, 26, 27]. Vital points were illustrated through the verbatim quotations from the FGDs and these were further controlled by the available literature. Trustworthiness was ensured through the four principles of Lincoln and Guba’s framework [28]. Credibility was ascertained by a prolonged engagement which increased rapport and with participants. Data triangulation was confirmed by using different data collection methods through field notes, in-depth individual interviews, referential adequacy and co-coder. Confirmability was ensured by audit trail of voice recorder and the field notes to determine that the conclusions, interpretations and recommendations were indeed traceable to the sources. Transferability was ensured through the thick description of the research methods and design.

## Results

The participants selected for his study were all NIMART trained nurses, of whom the majority were female (*n* = 17; 70.8%) and their age ranged from 24 to 58 years [24, 26]. The experience ranged between 3 and 4 years and they worked in both PHC (*n* = 11; 45.8%) and CHC (*n* = 13; 54.2%) [24, 26]. The two themes that emerged were positive attitudinal needs and positive behavioural change. Positive attitudinal needs incorporated the need for improved accessibility to the development and implementation of TB/HIV treatment guidelines, provision of motivation, support and supervision to NIMART trained nurses, the adaptation of NIMART trained nurses to practice change, and the improvement of knowledge and awareness among NIMART trained nurses. The positive behavioural change incorporated the organisational-structural changes, availability of the user-friendly guidelines and the anticipated patient responsiveness to the treatment guidelines. Table 1 summarises the themes and sub-themes that emerged from the analysed data [26].

**Table 1:** Themes and sub-themes as facilitators of adherence to treatment guidelines [26]

Sub-theme	Category
1.1 Positive attitudinal needs	1.1.1 The need for improved accessibility to the development and implementation of TB/HIV treatment guidelines
	1.1.2 The provision of motivation, support and supervision to NIMART trained nurses
	1.1.3 The adaptation of NIMART trained nurses to practice change
	1.1.4 The improvement of knowledge and awareness
1.2 Positive behavioural change	1.2.1 The organisational-structural changes
	1.2.2 The availability of the user-friendly guidelines
	1.2.3 The anticipated patient responsiveness to the treatment guidelines

### Factors facilitating treatment guidelines adherence among trained NIMART nurses’

The likelihood of enhanced TB/HIV treatment guidelines usage and adherence was upstretched in this study. The adherence to treatment guidelines could be improved as reported by the NIMART trained nurses. The findings provided insight in this regard and revealed that it is possible through the positive attitudinal needs and the positive behavioural change [26]. These two factors were viewed as factors that NIMART trained nurses perceived to have the potential to promote their level of treatment guidelines usage and adherence.

#### Positive attitudinal needs

The NIMART trained nurses identified and expressed the following needs, namely, the need for improved accessibility of TB/HIV treatment guidelines; provision of motivation, support and supervision to NIMART trained nurses; the adaptation of NIMART trained nurses to practice change and the improvement of knowledge and awareness among nurses [26].

##### The need for improved accessibility of TB/HIV treatment guidelines

NIMART trained nurses articulated that a basic and easy-to-use guideline (such as a handbook, pocketbook and/or flowcharts) has the potential to enhance the usage of TB/HIV treatment guidelines and it is through this that adherence can be promoted. The NIMART trained nurses verbalised that:

*“A portable guideline that can be accessible and owned by any health care provider can ease and promote adherence, not just one guideline for the clinic.”* (P9, FGD 4) [26]

*“If they can change just a little bit of the size so that it can be like a handbook that is easy to use and quick to go through.”* (P 4, FGD 1) [26]

##### The need for the provision of motivation, support and supervision to NIMART trained nurses

The findings of this study report that NIMART trained nurses do require some form of support in their caregiving role for them to utilise and adhere correctly and accurately to the treatment guidelines. It was established that NIMART trained nurses may well adhere better to treatment guidelines and yield better outcomes if they received adequate support and supervision. This was manifested in the following participant’s submission:

*“We need support and encouragement to carry out our nursing duties to the best of quality possible. Weekly or monthly supervision or support visits can increase the level of adherence and guidelines usage among nurses and this will promote the quality provision of care to our patients.”* (P 10, FGD 2) [26]

Though, NIMART trained nurses reported that if there is a positive working association between nurses and other allied health care providers, then all TB/HIV patients could be treated thoroughly with a marked advanced needed level of adherence. Participants uttered the following:

*“A good working relationship can promote adherence to treatment guidelines as not only nurses provide care to TB and HIV patients.”* (P 8, FGD 2) [26]

*“We are supposed to work hand-in-glove [hand in hand] with one another for the provision of quality care to our patients.”* (P 11, FGD 4) [26]

##### The need for adaptation of NIMART trained nurses to practice change

It was uttered that NIMART trained nurses do want to change, however, the health care system fails to permit them as the evident pressure of task-shifting continuously and constantly catches up. One NIMART trained nurse voiced the following:

*“The department of health should allow us, nurses, to move slowly as this was not our scope of practice to be well orientated, knowledgeable and skilful. The reason we don’t want to move from our past routines is that it takes time to acclimatise to the new things. I was trained for NIMART in 2011, but I still find it hard to fully understand the initiation and management of ART.”* (P 10, FGD 2) [26]

A steady change within the Department of Health’s health care system processes may present potential usefulness by providing NIMART trained nurses with enough time to adapt and accommodate the appropriate guideline changes in practice and within the TB & HIV service needs. Most NIMART trained nurses emphasized that they are not constantly in pace with the novelty and new developments in their practice, while there is marked reduction in their level of treatment guidelines usage and adherence. Essentially, a steady provision of orientation to the new developments in NIMART has the potential to promote and improve NIMART trained nurses’ adherence to treatment guidelines.

##### The need for improved knowledge and awareness among NIMART trained nurses

The need for adequate provision of orientation to new developments in treatment guidelines, guidelines updates (refresher courses), continuous training (including in-services) and follow-up training to NIMART nurses and additional practices have been identified as priorities that have the potential to increase the treatment guidelines adherence level. The NIMART trained nurses verbalised that:

*“I think more nurses need to be trained in NIMART or all nurses in each facility need to be trained as this causes gaps in the health care provision. Patients won’t be returned back [sic] because of a trained nurse not being available.”* (P 6, FGD 1) [26]

This sentiment accentuates the point on the provision of treatment guidelines training and education to promote usage and adherence. Furthermore, to substantiate this, there is a need for continuous follow-up training that should be done within the facilities that the NIMART trained nurses are working in as to avoid perpetuating the dearth of trained nurses. One NIMART trained nurse accentuated that:

*“We know it is impossible to train everyone in time, but if there is something new coming, even if it’s not a formal training but trainers can visit the facilities just to provide an insight on the available change while training is taking place.”* (P 4, FGD 3) [26]

### Positive Behavioural change

The focus group discussions revealed that behavioural changes can intensify and enhance treatment guidelines adherence level, particularly within the organisational-structural changes, availability of the user-friendly guidelines and the anticipated patient responsiveness to the treatment guidelines.

#### The organisational-structural changes

The organisational-structural changes included enough working time, ample or adequate human resources, acceptable and appropriate workload, proper communication between staff, management and patients, and the accessibility of the core treatment guidelines. If NIMART trained nurses are provided adequate time, then it would be very easy for them in making use of and adhering to TB/HIV guidelines. The NIMART trained nurses uttered that:

*“We need enough time to work with patients as well as to follow the guidelines correctly. Instead of the system pushing us to do more quantity meaning more headcount per day, it should provide time for us to provide quality care to our patients. There is no use for a patient to spend the prescribed 2 hours in the clinic and you find that no quality care provided to this patient.”* (P 1, FGD 1) [26]

*“Reduced workload and reduced time pressure can increase adherence to treatment guideline and also promote the provision of quality care to our TB and HIV patients.”* (P 8, FGD 2) [26]

*“We know it is impossible to train everyone in time but if there is something new coming, even if it’s not a formal training but trainers can visit the facilities just to provide an insight on the available change while training is taking place.”* (P 10, FGD 2) [26]

Participating NIMART trained nurses raised another strategy which has the potential to promote treatment guidelines adherence on NIMART trained nurses which involves availing the TB/HIV treatment guidelines within the facility. Thus, each consulting room in each facility must have accessible treatment guidelines. One NIMART trained nurse highlighted:

*“Guidelines need to be made available in the facilities for easy use and accessibility. However, not just one guideline, but enough for each health care provider as this will reduce the time for looking for a guideline or waiting for one to be done with it so that one can use it.”* (P 6, FGD 3) [26]

Nurses also voiced that proper communication channels could promote adherence to treatment guidelines. A participant spoke on this point in insightful ways:

*“Good communication between the implementers, programme managers, coordinators and supervisors can promote adherence to treatment guidelines. Any change needs to be communicated to the implementers’ way beforehand not just in the implementation phase.”* (P 7, FGD 4) [26]

#### The availability of the user-friendly guidelines

A simple, clear and non-complicated TB/HIV treatment guideline may improve the use and adherence level among NIMART trained nurses. One NIMART trained nurse stated the following point to endorse this observation:

*“A simple guideline that is clear and at the level of nurses can be of help to us as it will be easy to understand and go through. The chart or handbook or pocketbook will be of greater help. But I like that diagram like poster … Yes, the algorithm type of guidelines. It is simple and easy to follow rather than the book.”* (P 12, FGD 4) [26]

#### Patient responsiveness

The treatment guidelines adherence was also portrayed with dependency on the abilities of TB and HIV patients/clients to entirely participate in their ART and anti-TB treatment. Thus, the cooperation, availability, engagement and compliance of patients/clients can also improve the treatment guidelines adherence of NIMART trained nurses. Some of the NIMART trained nurses verbalised that:

*“Sometimes we need patients on board; we don’t monitor patients because they are not available or complying to the monthly visits that we set for them. If patients can adhere and follow all that we say to them this can ease our work and promote adherence to treatment guidelines.”* (P 5, FGD 3) [26]

*“I agree with you patients are our customers and their availability and engagement in the provision of ART and TB treatment can increase our adherence.”* (P 6, FGD 3) [26]

## Discussion

The NIMART nurses expressed the need for guidelines written in a simple and uncluttered style to allow and promote both content and message clarity. It was also emphasised that the size of the guideline should be manageable and portable to carry around. Literature also confirms that the treatment guidelines usage is enhanced when a nurse sees the usability of the treatment guideline in daily practice [19]. Furthermore, treatment guidelines could be implemented correctly and adhered to when they are easy to understand, straight-forward and user-friendly [29].

Nurses felt that there is a need to be supervised, motivated and supported in implementing treatment guidelines so that adherence can be promoted. The importance of constant supervision that is provided to NIMART nurses until they get used to correctly implementing treatment guidelines was raised across the cohort of participants in both provinces. This was affirmed by literature which specifies that physicians, programme managers and coordinators as well as facility supervisors’ support can significantly encourage NIMART nurses’ adherence to treatment guidelines [19]. Furthermore, the minute a good working relationship is present amongst healthcare providers, patients are cared for and managed way better and with adequate treatment guideline adherence level. Treatment guidelines adherence can be enhanced once there is lateral cooperation or teamwork among all healthcare providers. Any gap in the protocol of communication was perceived as an impediment and a reason for non-adherence identified [19, 29]. Thus, communication and good working relationships should be enhanced to promote adherence to guidelines. NIMART nurses reported that they are for change, unfortunately, they are overwhelmed by the system related changes as the pressure of task-shifting, continuous guidelines changes and developments in the management of TB/HIV over-stretches them as the programme implementers [26]. Quite often, the FGDs raised the point that these nurses are not consulted or engaged in those changes and developments that consequently affect them directly.

There is a sentiment that there should be a continuous provision of training about treatment guidelines implementation to promote adherence and usage. Participants also insisted that the introduction of new TB/HIV services ought to be gradual as nurses needed time to adjust to the novel changes [26]. Literature, as evidenced from other previous studies, highlights the element that most nurses recognize the significance of being offered orientation, necessary training and education concerning TB/HIV treatment guidelines as these promote treatment guidelines adherence and use [19, 29]. Additionally, there is a dire need for continuous in-service and follow-up training that are facility-based to prevent impact on the already suffering workforce to limit the shortage of NIMART trained nurses [26]. Another study emphasises the significance of using a trained person or outsourced team of NIMART trained nurses from external organisations to conduct educational outreach visits to all nurses within their facilities to share information, practices and materials with the intent of instilling the need for change the health care providers’ practise to help increase their level of knowledge [30].

The reduced workload promotes the smooth usage of and adherence to treatment guidelines. In instances where a NIMART trained nurse is faced with an adequate workload, it enables and promotes the usage of and adherence to treatment guidelines [19]. An appropriately trained nursing workforce (NIMART trained nurse), have the potential to promote the continuance and sustenance of quality health care provided in the CHC and PHC facilities and will continue to promote the adherence to treatment guidelines [26]. Noteworthy, the NIMART trained nurses could then be apportioned duties without overstretching the numbers and fairly focusing on the quality of care provided.

To promote adherence to and usage of the treatment guidelines, the treatment guidelines should be available in the consulting rooms of each CHC and PHC facilities [26]. Thus, the TB/HIV treatment guidelines must be accessible to all NIMART trained nurses who are tasked to initiate and manage TB and HIV. In support of the latter, the availability of treatment guidelines to the NIMART trained nurses has the potential to promote the usage of such guidelines [19]. The NIMART trained nurses further highlighted that adherence and usage of the treatment guidelines are also promoted using proper communication channels [26]. Appropriate and adequate communication amongst guideline implementers (NIMART trained nurses) and developers are essential given its ability to promote adherence and usage thereof [19, 30]. The user-friendliness of the TB/HIV treatment guidelines could promote adherence and usage. This viewpoint was borne out by different authors that treatment guidelines that are understandably clear and easy enjoy a higher chance to be utilised which at the end promotes adherence [19, 30, 31].

Also, the need for TB/HIV patients or clients’ full participation in their care and management have been highlighted as a potential facilitator for usage and adherence to treatment guidelines. This is normally observed when the patients/clients always avail themselves in the facility for all their set assessments, tests and monitoring, as well as their adherence to TB/HIV therapy, regimens and prescriptions [26]. However, it was also reported that the use of treatment guidelines could only be perceived as a lack of confidence in what the health care provider does [26]. Some studies suggest that guidelines are time-consuming [19, 30–34].

## Conclusion

The study has clarified different aspects that need to be addressed to facilitate the adherence to guidelines by NIMART trained nurses. Factors such as continuous TB/HIV related training, support supervision and improved relationships with managers and colleagues, all need to be provided, promoted and enhanced to reach the desired outcomes efficiently and effectively. Cumulatively, these aspects exert a positive impact on service delivery**.** It is recommended that the inclusion of NIMART trained nurses in the development of treatment guidelines may promote their use and adherence. Support supervision from TB/HIV programme supervisors and trainers should be made available constantly and debriefing sessions conducted with NIMART trained nurses regularly. Regular in-service training for all stakeholders should be implemented, in tandem with seminars or workshops with NIMART trained nurses designed to update them and offer refresher courses. Further research in this practice could evaluate improvements in the implementation of guidelines as well as the impact thereof.

## Data Availability

The datasets generated and analysed during the current study are not publicly available due to the nature of ethical approval which stated that only the research team had access to the collected data but are available from the corresponding author upon reasonable request.

## Abbreviations

ART: Antiretroviral Therapy
CD4: Cluster of Differentiation 4
CHC: Community Health Centre
FGD: Focus Group Discussion
HIV: Human Immunodeficiency Virus
KZN: Kwazulu-Natal
MDR-TB: Multi-Drug Resistant TB
NCT: Notice- Collect- Think
NIMART: Nurse-Initiated Management of Antiretroviral Therapy
NRF: National Research Foundation
NW: North West
NWP: North West Province
P: Participant
PHC: Primary Health Care
STIs: Sexually Transmitted Infections
TB: Tuberculosis
WHO: World Health Organisation
XDR-TB: Extensively drug-resistant TB
